# Development and validation of impact of early integration of palliative care and oncology(IEI PCO) questionnaire: a survey for medical oncologists and nurses

**DOI:** 10.1186/s12904-024-01435-1

**Published:** 2024-04-26

**Authors:** Abdulrahman Abdulaziz Abdullah, Wafaa Mostafa Abd-El-Gawad, Sobhi Mostafa AboSerea, Fatma AbdelShakor Ali, Saima Ali

**Affiliations:** 1 Consultant and Head of Palliative Medicine Department, Palliative Care Center, Kuwait, Kuwait; 2 Associate Professor of Geriatrics and Gerontology Medicine, Geriatrics and Gerontology Department,Faculty of Medicine,, Ain Shams University, Al-Abbaseya, Cairo, Egypt; 3 Palliative Medicine Department, Palliative Care Center, Kuwait , Kuwait; 4 Research Analyst, Division of Continuing Professional Development, Weill Cornell Medicine, Qatar, Qatar

**Keywords:** IEI PCO: impact of early integration of palliative care and oncology survey, PCO: Palliative care Integration, Kuwait, Oncology

## Abstract

**Objectives:**

Many associations have recently recommended early integration of oncology and palliative care for more standard cancer care and better quality of life. We aimed to create a questionnaire to assess the opinion of medical oncologists and nurses about the clinical impact of the integrated palliative care and oncology (PCO) program.

**Methods:**

A novel semi-structured questionnaire called Impact of Early Integration of Palliative Care Oncology (IEI PCO) questionnaire was developed and tested for validity and reliability then distributed to the oncologists and nurses working in Kuwait Cancer Control Center.

**Results:**

After the pilot stage, testing the final questionnaire for validity and reliability was done with satisfactory results. Finally, the complete questionnaires were 170 out of 256 (response rate 66.41%). More awareness about the available palliative care services and the new available PCO services (*p*-value < 0.001 for all). Most of the oncologists and nurses agreed with the currently available structure of PCO, appreciated the patients’ discharge plan and continuity of care of palliative medicine, admitted less work burden, a better attitude, and higher satisfaction (*p*-value for all < 0.001) toward palliative care. Significant improvements in symptoms were appreciated by oncologists and nurses after the integration of palliative care (*p*-value for all < 0.001. Oncologists and nurses valued repeated honest communication, discussion of the goals of care, dealing more effectively with ending active treatment, and higher acceptance of patients and families of PC policy of transfer, and significant progress in the care of end-of-life symptoms (*p*-value for all < 0.001).

**Conclusions:**

The IEI PCO questionnaire demonstrated the psychometric criteria for content, face, and construct validity and reliability. It provides a valuable tool to assess the impact of PCO integration. The opinion of medical oncologists and nurses was significantly positive toward the early integration of PCO in Kuwait in most aspects of care. This integration led to improved symptom control, end-of-life care, communication, and planned discharge and follow-up plans. Moreover, decreases the work burden, improves attitude, higher satisfaction of the oncology staff, and continuity of care.

**Supplementary Information:**

The online version contains supplementary material available at 10.1186/s12904-024-01435-1.

## Key message

**What is already known?**

Many studies proved the role of integrated palliative and oncology care but no available tools to assess this impact in clinical practice.

**What does this study add?**

• Impact of Early Integration of Palliative Care and Oncology Questionnaire (IEI PCO) is a newly developed and validated tool that can be used for evaluation of the impact of the PCO integration program.

• It compares symptom control, end-of-life care, professional communication, structure, and clinical practice of PCO before and after integration and how this can affect the work burden, attitude, and satisfaction of oncology staff.

**How this study might affect research, practice, or policy?**

• It increases the awareness of oncology staff about the available PCO models such as ambulatory SPC team. The joint clinic, weekends round by SPC?etc

• It can provide a guide to the important aspects that should be considered during the preparation of any PCO integration program and give feedback about aspects that need improvement.

**Supplementary Information:**

The online version contains supplementary material available at 10.1186/s12904-024-01435-1.

## Introduction

Palliative care (PC) is defined by the World Health Organization (WHO) as an approach that improves the quality of life of patients and their families facing the problems associated with life-threatening illnesses [1]. Traditionally, palliative care was labeled only for patients at the terminal stages [2] although it became evident that those patients may encounter physical psychological, and spiritual problems early in the disease course. These problems become clearer with the aging of the population, so more patients with incurable life-threatening illnesses and limited survival are in critical need of palliative care [3]. 

Recently, emerging evidence has shown significant benefits from the early introduction of palliative care in the management of this group of patients. These benefits included significantly better quality of life, less symptom burden [4], clear discharge and follow-up plan, active communication with the patients and families about diagnosis and prognosis [5–7], and a higher degree of satisfaction [8, 9] as well as the less aggressive end of life interventions [10, 11]. 

Since 2012, the National Comprehensive Cancer Care Network’s palliative care guidelines recommend screening of all patients for palliative care issues during initial oncology consultation and at clinically relevant times [12]. Thus WHO [1], the American Society of Clinical Oncology (ASCO), and the European Society for Medical Oncology (ESMO) have recently recommended early integration of oncology and palliative care [13–15] in inpatient and outpatient care together with anticancer treatment.

Recently, in Kuwait, the introduction of u (SPC) service was done through the standalone Palliative Care Center (PCC) in 2011but it is used to care for patients at the end of life. In September 2016, the palliative medicine department in PCC started to look after the symptoms of cancer patients in medical oncology wards at Kuwait Cancer Control Center (KCCC). Criteria for palliative care referral were settled and updated regularly (Appendix 1). Since then, the oncologists in KCCC have referred their patients early to the specialized palliative care (SPC) team for symptom control either as an inpatient or outpatient consultation. So, the PCO integration between SPC as an embedded service in KCCC and the oncology department was started. Measurement of the impact of this implementation on structure, process, different available models per center, and efficient function of this implementation was needed. Till now, to the best of our knowledge, no available tool to assess and measure these outcomes for further improvement of the PCO service.

### Objectives

A. Primary objectives.

1. To create a reliable and validated tool to measure the impact of early integration of palliative care and oncology (PCO).
2. To apply this validated and reliable tool on the oncology staff for further validation.

B. Secondary Objectives.

1. To view the opinion of medical oncologists and nurses about the structure of clinical practice and the process of care of the current integrated PCO program.
2. To view and compare the opinions of medical oncologists and nurses about the impact of early PCO on symptom management, communication with the patient and family, and end-of-life care before and after early integration.
3. To determine the impact of early referral to implementation of PCO on work burden, attitude, and satisfaction of medical oncologists and nurses.

### Patients and methods

#### Study design and setting

A novel semi-structured questionnaire was created through a multi-stage process of survey development to measure the opinion of medical oncologists and nurses about the impact of the integration of PCO. Measurement of the implementation of PCO integration regarding the structure, process, different available models, and efficient function aimed to ensure better symptom management, good communication with patient and family, end-of-life care, and how it would help to decrease the work burden, improved attitude and increases the satisfaction of the medical oncologists and nurses.

After the development and validation of the questionnaire, it was distributed in KCCC. All medical oncologists and nurses in the medical oncology department who had at least two years of experience working at the KCCC were invited to participate. From them at least one year before and one year after the start of the integration to ensure that all participants attended and witnessed the differences.

### Pilot study: development and evaluation of the preliminary questionnaire

#### Instrument design

Based on the current literature, unfortunately, there are limited validated tools to measure the actual clinical impact of early integration of specialist palliative care (SPC) in a tertiary cancer center. Determination of content domains was obtained from the literature review [8, 15–23]. Interview with the respondents and focus groups (Target population) and expert panel opinion. This aimed to give a clear image of the boundaries of the dimension components. Qualitative data collection from the above resources was done and then returned to the research question to ensure items were relevant to the question [24]. 

The focus group had four oncologists and nine nurses (target population) and the expert panel in the academic and clinical field of palliative care and oncology co-coordinators guided throughout the entire development process of the questionnaire. The membership of the expert panel included five consultants from palliative care, two medical oncology consultants, the head nurses of palliative medicine and oncology departments, and two professionals in psychometric analysis. The main clinical aspects needed to assess the impact of palliative care on oncology (PCO) were divided into seven main domains. The first main section was about the current structure of clinical practice and the process of care while other sections were about clinical domains such as a comparison of differences in symptom management, communication, and end-of-life care before and after at least one year of early integration of PCO and in turn how the improvement of these aspects affect work burden, the attitude, and satisfaction of oncology staff together with one section about the current structure of clinical practice and the process of care.

### Judgment

After a series of meetings and discussions with an expert panel and focus group, the items were refined to a pool of approximately 291 items covering all dimensions and organized in a suitable format and sequence in a usable form. Most of the items were identified and measured through a points Likert scale [25, 26]. In many studies, five points scale is comprehensible and helps to quantify subjective preferential opinion, attitude, thinking, and feeling accurately and in a scientifically accepted, validated, and reliable manner [26, 27]. Participants were asked to show their level of agreement [from strongly disagree (1) to strongly agree (5)] with the given statement (items) on a metric scale. So, all the statements in combination in each section should be interlinked with each other [26–30]. While some items were added in ‘yes or no’ form to be more decisive e.g., awareness about the availability of different PC services before and after PCO integration such as inpatient consultation and outpatient clinics, and one open-ended question about aspects of services that may need improvement.

Several questions were added about demographics, and palliative care education to characterize the respondents such as sex, age, any formal palliative care training…etc. Now, the preliminary instrument was then ready for piloting and included 124 items.

The questionnaire was distributed to palliative medicine physicians and oncology staff followed by qualitative and quantitative analysis.

### Evaluation of validity and reliability of the final questionnaire

The final questionnaire was distributed to oncology staff in KCCC (medical oncologists and nurses). Again, qualitative and quantitative analyses of the items were done. To measure the test-retest reliability [31, 32] of the final version, the questionnaire was administered on two separate occasions, with an interval of two weeks between them. Two weeks were expected to be long enough for participants to have forgotten their original responses, but not sufficiently long for much real change in their opinions about the impact of early integration of palliative care. To match the two sets of questionnaires, birth dates were used. The first set of questionnaires was used for construct validity and internal consistency.

The final validated tool is composed of 9 sections:

The first and second sections of the questionnaire included oncologists’ and nurses’ socio-demographic characteristics such as age, gender, qualifications (nurses and physicians), years of work experience, occupation, and unit/ ward (physicians and nurses), and prior palliative care education.

The third section inquired about the structure, models, and inpatient and outpatient consultation services of palliative care that should be existing for cancer patients and what is currently available compared to before PCO integration.

The fourth to sixth sections were evaluating the impact of PCO integration on the quality of care, including symptom management, communication with patients and their families, and end-of-life care. Participants were asked to rate their level of agreement with items both before and after the introduction of the PCO.

The seventh to ninth sections measured the impact of the PCO on the work burden, attitude, and satisfaction of the oncology staff. These all were measured using a 5-point Likert scale to measure the degree of agreement/satisfaction with different items.

Finally, one item enquired if participants found the PCO service could be improved (Yes/ No) and one open-ended question allowed space for participants to suggest any improvement. Development, Pilot study, and validation of the questionnaire took around one and a half years to finalize the survey.

### Ethical consideration

The approval of the ethical committee of the Ministry of Health was obtained (2017/62). The aim of the study and expected outcomes were discussed with all participants including the expert panel and focus group guaranteeing the privacy of the data and written informed consent was obtained from them. Permission from the KCCC Director and heads of oncology and nursing departments in KCCC was also taken.

### Statistical analysis

Revision of the raw data, coding of the data, and master tables preparation were done. Data entry, manipulation, and analysis were done on SPSS (Statistical Package for Social Science), Version 20.

### Development and evaluation of the preliminary questionnaire

Content validity was confirmed by the expert panel and the focus group (target population). Moreover, they tested and checked for relevancy, clarity, comprehensiveness, and representation. Content validity was assessed qualitatively for grammar, proper wording, order, and scoring and quantitively content validity Ratio (CVR) for each item. Item content validity Index (I-CVI) and scale content validity index (S-CVI) were also calculated then the instrument items were checked for comprehensiveness [29, 30, 33–35]. These repeated three rounds to ensure the highest content validity.

To ensure high face validity and the representation of a reasonably valid sample of items from the substantive areas of interest, items were then reviewed by all members of the expert panel and the focus group to select the best [29, 30, 35]. 

The results of the pilot study were analyzed for item discrimination and internal consistency and qualitatively. For item discrimination, Pearson correlation was used to compare each item with its subtotal score. Most literature chooses < 0.2 as a cut point for an item-to-total-score correlation to be rejected due to the inability to discriminate different answers related to this section during testing [27, 28], and to make it more relevant we retained only the items that met the correlation of > 0.3 or higher. Internal consistency using Cronbach’s alpha was measured separately for the different sections and 0.6 or more are accepted for research purposes [28, 36]. While the qualitative analysis was done by careful revision of each comment made by respondents. Repeated factor analyses (exploratory and confirmatory) were done throughout the study to help detect the number of the components’ domains and the correlations between items in each domain and between different domains.

### Validation of the final questionnaire “Impact of early integration of palliative care and oncology’’(IEI PCO survey)

The results of the final survey were tested again for construct validity, internal consistency, and Test-retest reliability. Unfortunately, testing of construct validity [31, 32] of the final version needed to be administered into two groups known to differ in their level of impact of PCO integration, and this PCO integration is the first one held in Kuwait either with cancer or any other specialty. So, we decided to compare the two responses for the same participant as before and after integration nevertheless, this was only done for four sections.

Testing for item normality by Shapiro-Wilk and Kolmogorov-Smirnov tests was done. For comparing two repeated measurements for the same group Wilcoxon Signed Ranks test for non-parametric items while the Paired t-test for parametric items was used when appropriate. The chi-square test or Fisher’s Exact test was used when appropriate to compare quantitative and qualitative variables respectively. Cronbach’s alpha was used to test internal consistency as mentioned above. Test-retest reliability was done to verify that the results produced were consistent over time. The intraclass correlation coefficient (ICC) test was used to compare the response of the same group before and after two weeks. More than 0.8 was considered an accepted cut-off point for reliability and consistency over time.

Qualitative analysis of the open-ended question at the end of the survey was done through inductive thematic analysis for quantitative data to identify the main themes through analysis of the responses of the participants.

Likert scale items scored as continuous/interval and the score of each construct was summed together and divided by the number of 5-point Likert scale items then the sum of each construct was collected. The questionnaire is divided into two main parts; structural (section III) and clinical part (sections IV to IX). Scoring of structural domains (section III) (21 items) scored from 2 to 21 points. Q1 and Q6 were divided by the number of the items (5 points Likert scale), and Q2 (yes = 1 after integration) scored from 0 to 8 points according to awareness of the availability of PCO integration service at the respondent’s center. Q3, Q4, and Q5 scored then divided by 3 so the maximum points were 3. So, the difference (19 points) was divided into three unequal parts based on weighted scoring during factor analysis. Poor structural PCO integration scored < 7, incomplete structural PCO integration ≥ 7 to < 13, and Accepted/Good structural integration ≥ 13 to 21 points.

Scoring of clinical domains (section IV to IX: 42 items): the score ranged from 6 to 30, so, the difference (24 points) was divided into three equal parts for scoring. Poor clinical PCO integration scored < 14, incomplete clinical PCO integration ≥ 14 to < 22, Accepted/Good clinical integration ≥ 22 to 30 points. The total score (min 8 to max 51) was divided as follows; poor PCO integration scored < 21, incomplete PCO integration ≥ 21 to < 35, and Accepted/Good integration ≥ 35 to 51 points.

## Results

### Development and evaluation of the preliminary questionnaire: 124 items

Qualitative content analysis was done as mentioned before. The expert panel requested thrice to judge content validity, CVR, and CVI. Moreover, they assessed for instrument comprehensiveness based on the construct of the underlying study [29, 33–36]. CVR of more than 0.59 based on the Lawshe Table [37] according to expert numbers were considered valid. Item content validity Index (I-CVI) > 79% considered valid, 70–79% needs revision and < 70% should be eliminated scale content validity index (S-CVI) was also calculated and then checked for items comprehensiveness [29, 30, 33–35]. 

First round of judgment, 104 items were removed because they had CVR < 0.59. The remaining items based on content experts were modified, and overlapped items were removed. The remaining items were 172 items. (Table 1S, 2S) Second round, CVR, I-CVI, and S-CVI for judgment of relevancy and clarity were calculated. 14 items were removed for low CVR (< 0.59) 27 items were removed for low CVI (< 70%) and 16 items were modified for intermediate CVI. After modification, 125 items remained. They discussed with the expert panel member for the third time for relevancy, clarity, and comprehensiveness for each dimension of the construct underlying the study.

After their judgment, face validity was tested by a sample of the respondents together with the members of the expert panel to select the best in terms of its importance, clarity, apparent attractiveness and relevance of the questions to the research objectives, and interpretability [24, 38, 39]. This process reduced the number of items to 109 as a preliminary questionnaire ready for piloting.

Most of the expert panel members participated in all meetings till the end of the pilot stage of the study. This stage took around one year and expert panel meetings were held bimonthly till the pilot stage finished.

Of one hundred and eight questionnaires distributed, the response rate was 75.93% and 82 questionnaires were returned and completed. Males were 39% (*n* = 32), their mean age was 37.13 (7.41), oncologists were 31.7% (*n* = 26) and nurses were 68.3% (*n* = 56). Item discrimination was tested for each section separately from section III to section IX (Table 1).

**Table 1:** Item Analysis for preliminary “Impact of Early Integration of Palliative Care and Oncology Survey’’(IEI PCO survey):

		Item no. before	Item no. after	Why	Item to sub-score correlation		Cronbach’s Alpha
					Minimum	Maximum	
Section III	1) Structure of PCO	9	6	Low item discrimination/ Poor internal consistency	0.767	0.947	0.811
	2) Simultaneous Oncology and PC	9	8	Repetition			
	3) and 4)Inpatient and Outside Care	7	3	Missed or irrelevant answers			
	5) Discharge	7	4	Low item discrimination/ Poor internal consistency	0.375	0.744	0.603
Section IV: Symptom Control	Before	11	9	Low item discrimination/ Poor internal consistency	0.319	0.777	0.730
	After				0.307	0.786	0.713
Section V: Communication skills	Before	8	5	Low item discrimination/ Poor internal consistency	0.309	0.811	0.764
	After				0.322	0.720	0.765
Section VI: End of Life Care	Before	10	7	Low item discrimination/ Poor internal consistency	0.364	0.847	0.685
	After				0.357	0.666	0.732
Section VII:	Work burden	9	7	Low item discrimination/ Poor internal consistency	0.409	0.820	0.732
Section VIII:	Attitude	16	7	Low item discrimination/ Poor internal consistency	0.312	0.678	0.688
Section IX:	Satisfaction	15	8	Low item discrimination/ Poor internal consistency	0.370	0.805	0.734

For item discrimination, 30 items were excluded due to poor discrimination (*r* < 0.3). Other item-to-total-score correlations (r) were ranging from 0.307 up to 0.947. Details are mentioned in Table 1. Furthermore, all items had a statistically significant relationship with the subtotal score (p-value < 0.05). Internal consistency was measured separately for the different sections using Cronbach’s alpha. It was acceptable ranging from 0.603 to 0.8. The main comment was the length of the questionnaire so some of the less relevant items in sections I and II (Demographic data and PC education) were removed to reduce the length of the questionnaire. Some changes to the wording were made in response to comments written on the questionnaires, to reduce uncertainty and maximize the clarity of the questions. Items with missed responses by most of the participants were also removed after missing data analysis testing in SPSS and then tested for the possibility of imputation process by using missing completed at random test (MCAR). This process of analysis leads to the dropping of nearly one-third (*n* = 48) of the original items. (Table 1)

### Validation of the final questionnaire “Impact of early integration of palliative care and oncology’’(IEI PCO survey): 76 items

The compliance was acceptable (66.41%). Distributed questionnaires were 256, and the completed ones were 170 so the response rate was 66.41%. The total number of oncologists who completed the survey was 39 (22.9%) and nurses were 131(77.1%). (Supplementary file: Fig. 1S)

Based on the analysis described above, the number of items was reduced to 76. The test to retest using intraclass correlation coefficient for testing reliability for the item was very high, ranging from 0.823 to 0.986 and the overall reliability was 0.91. Item analysis and internal consistency of the final survey were similar to the preliminary one. The internal consistency reliability of each section was established using Cronbach’s alpha (0.673 to 0.978). Item-to-sub-score correlations were very good for most of the items > 0.50 to 0.940. (Table 2)

**Table 2:** Item Analysis for “Impact of Early Integration of Palliative Care and Oncology Survey’’(IEI PCO survey)

			Item to sub-score correlation		Cronbach’s Alpha	Cronbach’s Alpha Based on Standardized Items
			Minimum	Maximum		
Section III	4) Structure of PCO	Doctors	0.924	0.940	0.817	0.978
		Nurses	0.731	0.885	0.805	0.941
		***Total***	0.775	0.896	0.809	0.952
	6) Discharge	Doctors	0.614	0.796	0.781	0.830
		Nurses	0.579	0.786	0.773	0.815
		***Total***	0.588	0.788	0.775	0.819
Section IV: Symptom Control	Before	Doctors	0.557	0.876	0.779	0.928
		Nurses	0.660	0.844	0.776	0.944
		***Total***	0.701	0.837	0.780	0.945
	After	Doctors	0.540	0.813	0.762	0.906
		Nurses	0.607	0.844	0.777	0.933
		***Total***	0.591	0.836	0.773	0.924
Section V: Communication skills	Before	Doctors	0.684	0.805	0.797	0.890
		Nurses	0.851	0.935	0.827	0.966
		***Total***	0.829	0.901	0.822	0.951
	After	Doctors	0.489	0.803	0.755	0.825
		Nurses	0.707	0.879	0.805	0.910
		***Total***	0.652	0.866	0.797	0.890
Section VI: End of Life Care	Before	Doctors	0.634	0.817	0.787	0.914
		Nurses	0.237	0.777	0.679	0.779
		***Total***	0.255	0.739	0.698	0.794
	After	Doctors	0.734	0.848	0.784	0.924
		Nurses	0.470	0.718	0.745	0.808
		***Total***	0.404	0.724	0.748	0.827
Section VII: Work burden		Doctors	0.534	0.762	0.770	0.881
		Nurses	0.505	0.791	0.761	0.849
		***Total***	0.505	0.758	0.765	0.859
Section VIII: Attitude		Doctors	0.223	0.842	0.675	0.651
		Nurses	0.231	0.661	0.673	0.680
		***Total***	0.200	0.675	0.675	0.682
Section IX: Satisfaction		Doctors	0.715	0.924	0.800	0.953
		Nurses	0.417	0.906	0.790	0.922
		***Total***	0.488	0.909	0.792	0.927

In the final factor analysis, the Kaiser-Meyer-Olkin Measure of Sampling Adequacy was > 0.7 for each component’s items and 0.702 for all items in all components together. Bartlett’s Test of Sphericity was significant (p value < 0.01) for each component’s items and all items in all components together.

The mean total score of the structural domains was 16.02 ± 2.13 points, clinical domains were 24 ± 1.78, and total PCO integration was 40.1 ± 2.9 points. This was denoting good structural and clinical PCO integration in KCCC.

The differences in demographic characteristics, palliative care education, and PCO models between oncologists and nurses are shown in Table 3. After PCO integration, all oncologists and nurses agreed that all or most of the patients were seen on the same day of inpatient consultation while in outpatient consultations, 66.6% (*n* = 26) of oncologists and 72.5% (*n* = 95) of nurses agreed that most the patients were seen on the same day.

**Table 3:** Differences in Demographic characteristics and Palliative care education between oncologists and nurses

			Oncologists *N* = 39	Nurses *N* = 131	P value
Age			40 ± 6.67	36.64 ± 6.86	0.008
Sex	Male		22 (56.4%)	32 (24.45)	< 0.001
	Female		17 (43.6%)	99 (75.6%)	
Occupation	Ass Registrar		2 (5.1%)	00	< 0.001
	Registrar		17 (43.6%)	110 (84.6%)	
	Senior Registrar		8 (20.5%)	14 (10.6%)	
	Specialist-consultant		12 (30.8%)	7 (5.4%)	
Years of experience			14.17 ± 6.24	11.5 ± 6.18	0.024
How much of your practice involves the care of patients with advanced (incurable) cancer?	Small proportion		6 (15.4%)	10(7.6%)	
	A substantial proportion		13(33.3%)	42(32.1%)	0.308
	Most of my practice		20(51.3%)	79(60.3%)	
PC Training			17 (43.6%)	48 (36.6%)	0.457
Duration of PC Training	≤ one week		8 (20.5%)	44(33.6%)	
	One month		4(10.3%)	2 (1.5%)	0.001^*^
	≥ six weeks		5(12.8%)	2 (1.5%)	
Relevance of PC training			18 (46.2%)	44 (33.8%)	0.003^*^
Available PCO models	Inpatient PC consultation service	Before	3 (8.1%)	14 (10.7%)	0.767^*^
		After	39(100%)	117(89.3%)	0.041^*^
	Regular palliative care outpatient clinic	Before	4 (10.3%)	11 (8.4%)	0.747^*^
		After	22 (56.4%)	90(68.7%)	0.180
	On-demand joint oncology-palliative care outpatient clinic	Before	12(31.65%)	14 (10.7%)	0.003
		After	38(97.4%)	118(90.1%)	0.194^*^
	Palliative care unit	Before	1 (2.6%)	2 (1.5%)	0.537^*^
		After	5 (12.8%)	81 (61.8%)	< 0.001
	Weekends and holidays PMT inpatient ward round	Before	0	0	00
		After	35 (89.7%)	117(89.3%)	0.999
	24/7 phone calls for continuity of care	Before	0	0	00
		After	23 (59%)	74(56.5%)	0.855
	Standalone PCC with 24 h services	Before	12(30.8%)	35(26.7%)	0.684
		After	23(59.0%)	74(56.5%)	0.855
	Community-based palliative care or home health care	Before	0	2(1.5%)	0.999^*^
		After	4 (10.3%)	43(32.8%)	0.007
Does your Centre have a dedicated PMT consultation service?			35(89.7%)	119(90.8%)	0.764^*^
Is the patient seen in the same day upon inpatient consultation?		All the time	4(10.3%)	20(15.3%)	0.602
		Mostly	35(89.7%)	111(84.7%)	
Is the patient seen in same day in the OPD upon demand of the oncologists?		All the time	2 (5.1%)	6 (4.6%)	
		Mostly	24(61.5%)	89(67.9%)	0.842^*^
		Rarely	9 (23.1%)	24(18.3%)	
		Don’t know	4 (10.3%)	12(9.2%)	

^*^ Fisher test was used

There were statistically significant differences between oncologists and nurses separately in overall sub-scores of symptom control, communication, and end-of-life care before and after PCO integration (p-value for all < 0.001) (Supplementary File: Table 3S, Fig. 2S). Moreover, there were also statistically significant differences in the response of each item before and after PCO integration in the models of delivery, symptom control, communication, and in the end of life care (p-value for all < 0.001) This met the criterion of construct validity. (Table 4)

**Table 4:** The differences between models for the delivery of PCO, symptomatic management, communication with patients and family and end-of-life care before and after PCO integration

	Mean	SD	Z	P value
**III. What are models for the delivery of simultaneous oncology and PC at your oncology center?**				
a) Inpatient PC consultation service	0.81548	0.52071	-10.934	< 0.001
b) Regular palliative care outpatient clinic	0.57396	0.55273	-9.377	< 0.001
c) On-demand joint oncology-palliative care outpatient clinic	0.76331	0.51485	-10.788	< 0.001
d) Palliative care unit	0.89412	0.30860	-8.899	< 0.001
e) Weekends and holidays PMT inpatient ward round	0.57059	0.49645	-12.329	< 0.001
f) 24/7 phone calls for continuity of care	0.29412	0.45699	-9.849	< 0.001
g) Standalone PCC with 24 h services	0.26627	0.46941	-7.071	< 0.001
h) Community-based palliative care or home health care	0.49112	0.52461	-6.429	< 0.001
**IV. Do you agree with the following statements about the symptomatic management of cancer patients before and after PCO integration?**				
a) Physical, psychological, social and spiritual pain was properly managed	1.26471	1.12815	-9.681	< 0.001
b) Dyspnea and other respiratory symptoms were easy to manage	1.08824	1.14529	-9.069	< 0.001
c) Difficult cases of nausea and vomiting were well controlled	0.92941	1.15424	-8.159	< 0.001
d) Constipation and other GIT symptoms were underestimated and under treated	0.92353	1.35438	-7.401	< 0.001
e) Psychological issues (e.g. depression, insomnia and anxiety) were routinely assessed and properly managed	1.22353	1.18048	-9.349	< 0.001
f) Delirium was easily identified and managed	1.08235	1.28452	-8.432	< 0.001
g) Opioids initiation, titration, rotation and related side effects were properly managed	1.38235	1.92484	-9.683	< 0.001
h) Symptoms were adequately controlled on discharge	1.08235	1.15857	-9.026	< 0.001
i) Allowing for more effective delivery of oncological treatments through control of symptoms	1.00000	1.18671	-8.331	< 0.001
**V. Do you agree with the following statements about communication with patients and family before and after PCO integration?**				
a) Repeated honest and accurate communication in a sensitive manner.	0.98824	1.11466	-8.699	< 0.001
b) Goals of care were discussed.	0.87647	1.05023	-8.171	< 0.001
c) Dealing more effectively with issues of ending active treatments.	0.90000	1.06393	-8.525	< 0.001
d) Conflicts among patient, family and medical team were resolved	0.81765	1.13926	-7.344	< 0.001
e) Higher patients’ and families’ acceptance of PC policy of transfer.	0.75294	0.99592	-7.593	< 0.001
**VI. Do you agree with the following statements about end-of-life care before and after the PCO?**				
a) End of life symptoms were effectively managed (e.g. delirium, pain, upper respiratory secretions)	1.27059	1.22487	-9.419	< 0.001
b) Prognosis was communicated clearly to the family.	0.78235	3.31301	-8.473	< 0.001
c) Compassionate communication was regularly delivered to patient, family and medical staff	0.87647	1.00415	-8.582	< 0.001
d) Bereavement support was provided	2.12941	1.22873	-10.610	< 0.001
e) Limitation of the role of life sustaining measures were discussed	1.62941	0.99005	-10.591	< 0.001
f) Patient and family values, preferences and goals were discussed and incorporated into PC plan	1.45882	0.89115	-10.720	< 0.001
g) Managing the place of death based on patient/family preference were discussed and declared (e.g.: ICU, home.)	0.81765	1.06961	-7.810	< 0.001

*Mean* of the differences of each item before and after PCO

After PCO integration, more awareness about the already available PCC services such as standalone PCC with 24-hour service and outpatient clinic (Z score − 7.071, -9.377, P-value < 0.001 for both) and the new PCO services such as inpatients consultation services, on-demand joint oncology-palliative care outpatientclinic, weekends and holidays, SPC inpatient ward rounds, and 24/7 phone calls for continuity of care (Z score − 10.934, -10.788, -12.329, -9.849, *p*-value < 0.001 for all).

Significant improvements in symptoms were appreciated by oncologists and nurses regarding the management of total pain, dyspnea, nausea and vomiting, constipation and other GIT symptoms, psychological issues, delirium, and opioids use (Z scores: -9.681, -9.069, -8.159, -7.401, -9.349, -8.432,-9.683, *p*-value for all < 0.001). Oncologists and nurses valued repeated honest communication, discussion of the goals of care, dealing more effectively with ending active treatment, and higher acceptance of patients and families of palliative care policy of transfer (Z score: -8.699, -8.171, -8.525, -7.593, *p*-value for all < 0.001). Significant progress in the care of end-of-life symptoms, clear compassionate communication about the patient’s condition and prognosis with the family, limitation of the role of life-sustaining treatment, and managing patients and family wishes including the place of death were esteemed by oncologists and nurses ( Z score: -9.419, -8.473, -8.582, -10.591, 10.721,-7.810, *p*-value for all < 0.001) (Table 4).

Most of the oncology staff agreed to the current structure and the process of clinical care of PCO, appreciated the discharge plan and continuity of care, admitted less work burden, a better attitude, and higher satisfaction about the role of palliative care in most items. (Table 5)

**Table 5:** Differences in the awareness about the structure and clinical process of PCO, continuity of care, work burden and satisfaction after PCO between oncologists and nurses

		Oncologists *N* = 39		Nurses *N* = 131	P value
**Do you agree with the following statements about the structure of the process of care of PCO?**					
All cancer centers must have PC services.	Strongly agree/agree	37(94.9%)		127(96.9%)	0.122*
	Strongly disagree/disagree	2(5.1%)		3(2.3%)	
Cancer patients should be seen by PMT even if they are on anti-tumor therapies.	Strongly agree/agree	31(88.4%)		115(87.8%)	< 0.001
	Strongly disagree/disagree	6(15.4%)		8(6.1%)	
Integrating all units of oncology with PC services has great impact on overall patients’ care and QoL.	Strongly agree/agree	34(91.3%)		122(93.1%)	< 0.001*
	Strongly disagree/disagree	2(5.1%)		3(2.3%)	
Process of PCO integration should take place in a structured way through departmental organizations, regular meeting and cases discussion	Strongly agree/agree	37(94.9%)		123(93.9%)	0.028*
	Strongly disagree/disagree	2(5.1%)		4(4.2%)	
Professional communication between oncology staff and PMT is essential for patient’ care.	Strongly agree/agree	34(91.3%)		115(87.8%)	< 0.001
	Strongly disagree/disagree	6(15.4%)		9(6.9%)	
Case discussion between PMT and oncologists increased oncologists’ experience in holistic care.	Strongly agree/agree	34(91.3%)		121(92.4%)	< 0.001*
	Strongly disagree/disagree	2(5.1%)		3(2.3%)	
**Regarding discharge planning and continuity of care**					
Adequate quantities of symptom control medications provided during discharge	Strongly agree/agree	39(100%)		131(100%)	0.832*
	Strongly disagree/disagree	00		00	
Follow-up plan provided during discharge	Strongly agree/agree	37(94.9%)		125(95.4%)	0.736
	Strongly disagree/disagree	00		00	
After hours support provide	Strongly agree/agree	36(92.3%)		118(90%)	0.922*
	Strongly disagree/disagree	2(5.1%)		13(9.9%)	
Preferred place of care discussed and facilitated	Strongly agree/agree	35(89.7%)		119(90.8%)	0.743*
	Strongly disagree/disagree	00		00	
**Do you agree with the following statements regarding work burden after PCO integration?**					
The length of oncologists’ visits to patients during rounds is reduced	Strongly agree/agree		30 (76.9%)	74(56.5%)	0.005
	Strongly disagree/disagree		7(17.9%)	47(35.9%)	
Number of patients’ calls are less	Strongly agree/agree		29(74.3%)	75(57.3%)	0.048
	Strongly disagree/disagree		5(12.7%)	48(36.7%)	
Number of nurses’ calls to the oncologists are less	Strongly agree/agree		28(71.8%)	114(87%)	0.097*
	Strongly disagree/disagree		4(10.3%)	11(8.4%)	
Number of patients’ visits to causality are less	Strongly agree/agree		25(64.1%)	46(35.3%)	< 0.001
	Strongly disagree/disagree		5(12.9%)	8(6.2%)	
Number of psychiatric and ICU consultations are less	Strongly agree/agree		27(69.2%)	77(58.7%)	0.001*
	Strongly disagree/disagree		3(7.7%)	25(19.1%)	
Duty hours became less stressful	Strongly agree/agree		27(69.2%)	69(52.7%)	< 0.001
	Strongly disagree/disagree		1(2.6%)	51(38.9%)	
I became more confident in dealing with patients’ symptoms	Strongly agree/agree		38(97.4%)	118(90%)	0.700*
	Strongly disagree/disagree		1(2.6%)	8(6.2%)	
**Do you agree with the following statements about the role of PC?**					
I likely to refer my patient to PMT when cancer is first diagnosed.	Strongly agree/agree		14(35.9%)	93(71%)	< 0.001
	Strongly disagree/disagree		24(61.6%)	32(24.45%)	
I have an ethical obligation to provide EoL care to my patient with terminal cancer rather than PMT.	Strongly agree/agree		25(89.7%)	124(94.75%)	0.003*
	Strongly disagree/disagree		3(7.7%)	7(5.4%)	
I only refer my patient to PCC at the time of impending death	Strongly agree/agree		7(18%)	47(35.8%)	0.222
	Strongly disagree/disagree		32(82.1%)	84(64.1%)	
Referring my patient to PMT makes me lose hope	Strongly agree/agree		6(15.4%)	46(35.2%)	0.012
	Strongly disagree/disagree		32(82.1%)	72(54.9%)	
I believe the response of PMT to referrals is slow.	Strongly agree/agree		9(23%)	30(22.95%)	
	Strongly disagree/disagree		28(71.5%)	95(72.5%)	
I think the criteria of PC referral is so restrictive to meet my patient’ needs.	Strongly agree/agree		30(82.9%)	103(78.6%)	0.967
	Strongly disagree/disagree		9(23.1%)	28(21.4%)	
I believe there is a need to educate patients, caregivers and even healthcare providers about the potential benefits of PC	Strongly agree/agree		25(64.1%)	123(93.9%)	< 0.001
	Strongly disagree/disagree		12(30.7%)	8(6.1%)	
**To what extent are you satisfied with ….?**					
Availability of PC services	Very satisfied/ satisfied		36(92.35%)	112(85.5%)	0.224*
	Very dissatisfied/dissatisfied		0	7(5.4%)	
Accessibility of PC services	Very satisfied/ satisfied		35(89.7%)	118(83.2%)	0.083*
	Very dissatisfied/dissatisfied		4(10.3%)	22(16.8%)	
Acceptability of PC services	Very satisfied/ satisfied		34(87.2%)	107(81.7%)	0.328*
	Very dissatisfied/dissatisfied		0	0	
Continuity of PC services	Very satisfied/ satisfied		34(87.2%)	114(87.1%)	0.503
	Very dissatisfied/dissatisfied		1(2.6%)	1(0.8%)	
Quality of PC services	Very satisfied/ satisfied		33(84.6%)	115(87.8%)	0.096
	Very dissatisfied/dissatisfied		6(14.4%)	16(12.2%)	
Cost impact of PC services	Very satisfied/ satisfied		27(69.2%)	115(87.8%)	0.045
	Very dissatisfied/dissatisfied		3(7.7%)	3(2.3%)	
The overall services provided by PMT	Very satisfied/ satisfied		37(94.9%)	119(90.8%)	0.112
	Very dissatisfied/dissatisfied		0	3(2.3%)	

^*^ Fisher test was used

Qualitative analysis of 84 participants responded to the open-ended question at the end of the survey. This was done through inductive thematic analysis. Participants replied positively craving for improving PCO integration. The thematic analysis yielded 3 predominant themes: the need for a regular Specialized Palliative Care (SPC) clinic in KCCC, a palliative care unit embedded in KCCC, and a regular palliative care education program annually or biannually.

## Discussion

No one can deny the role of early palliative care in the trajectory of life-threatening illness, especially with a new era of a rapidly aging population worldwide that is associated with an increased incidence of cancer [40, 41]. Given advancing medicine and more and more disease-modifying therapies worldwide, it becomes difficult for a single oncologist to manage all aspects of cancer care such as diagnosis, disease-modifying therapies, symptom control, and end-of-life care [41]. More clinical support is needed through PCO integration during the entire disease journey, not only for alleviating symptom burden but helping the oncologists achieve a high standard of cancer care [31]. 

To our knowledge, no available tools to describe the impact of PCO integration on different aspects of patient care together such as symptom control, end-of-life care, and communication, and how these aspects affect oncology staff regarding work burden, attitude, and satisfaction. A few questionnaires were used to measure a few aspects of the impact of early PCO integration such as those used in Salins et al. [8] and Zagonel et al. [15] studies. In Salins et study, the authors used a survey questionnaire that was not validated to know the role of early specialized palliative care (SPC) on symptom control, communication, and health-related communication only [8] while in Zagonel et al. study, authors created and validated a questionnaire that focuses mainly on different aspects of communication, training, research, and organizational models [15]. The new IEI PCO questionnaire focused on nearly all clinical aspects of patient care. Validation of the questionnaire was done through both a pilot study and testing of the final questionnaire. The final Survey results are similar to the preliminary one in terms of item analysis, internal consistency, item to subs-score correlation.

In our study, oncologists and nurses perceived PCO integration of palliative care as a positive experience regarding all aspects of cancer care such as symptom control, end-of-life care, and communication when comparing these impacts of PCO before and after integration. While; after PCO integration; they admitted less work burden, a better attitude, and higher degrees of satisfaction toward palliative care. Moreover, oncologists and nurses found that the specialized palliative care (SPC) team smoothed planned discharge, clear follow-up plans, and overall better continuity of care.

Many studies were conducted on the co-management model of early involvement of specialized palliative care (SPC) in oncology care helped the patients in many aspects. Better symptom control outcomes in the group under palliative care were proved in two randomized clinical trials [32, 42] and other studies [8, 43]. 

Another aspect of improved care is better end-of-life care and communication. Clinical studies showed that early referral provides room for the therapeutic relationship between the specialized palliative care (SPC) team and patients and families that allows discussion of sensitive issues such as cessation of disease-directed treatment, changing goals of care, diagnosis and prognosis, and better management of end of life issues [4, 8, 44, 45]. As the oncologists often hesitate to discuss these sensitive issues this may make the patients and their families depressed and lose hope.

According to the literature, the timing of simultaneous specialized palliative care (SPC) initiation is unclear [46]. Many of these “red flags” concern severe or ongoing symptoms, or other emergency admissions as reasons for SPC initiation [4]. Also, the cancer specialist is considered the gatekeeper of the initiation of concurrent SPC [25, 47, 48]. To date, still many oncologists would be uncomfortable initiating SPC “gold standard”, although the triggers recommended by ASCO [15] give valuable guidance. Finally; as outlined by the Lancet Oncology Commission; [40] the timing of PCO integration has to be tailored according to each cancer center’s needs after thorough interdisciplinary discussion.

Currently, the criteria of referral “triggers” to our specialized palliative care (SPC) in Kuwait is outlined in Appendix (1) but moderate to severe symptoms and frequent admission are at the top of the list for referral to SPC.

The availability of different models of PCO integration and prompt response of specialized palliative care (SPC) helps to deliver high-quality service for cancer patients in Kuwait. Currently, still, community-based palliative care (home care) is newly started by non-governmental organizations but is not well established. Few patients are covered by that service.

The last report published about the mapping of palliative care worldwide described the situation in 2017 and labeled Kuwait at the 3a level as an isolated palliative care provision [49] but currently; since integration in 2016, the situation much improved. Moreover, the Kuwait Cancer Control Center (KCCC) was categorized as ESMO-DC (Designated center) for PCO in 2017.

### Limitations of this study

This newly developed Likert-based questionnaire assesses the opinions of oncologists and nurses about the impact of PCO integration on different structural and clinical aspects of cancer care hence improving these services for better care. It is the first of its kind to measure the impact of PCO integration worldwide. This questionnaire was developed, validated, and tested for reliability through a pilot study and the application of the final questionnaire. However, the questionnaire is missing an assessment of integrated research and education programs and this is mainly due to the length of the questionnaire so we preferred to focus on the impact on clinical care as the first step before education and research.

Although a small sample size, this was considered representative because it included all available medical oncologists and nurses involved in the care of cancer patients in Kuwait.

## Conclusion

The IEI PCO questionnaire demonstrates the psychometric criteria for content, face, and construct validity and reliability. It provides a valuable tool to assess the impact of PCO integration. The opinion of medical oncologists and nurses was significantly positive toward the early integration of PCO in Kuwait in most aspects of care. This integration led to improved symptom control, end-of-life care, communication, and planned discharge and follow-up plans. Moreover, decreases the work burden, improves attitude, higher satisfaction of the oncology staff, and continuity of care.

### Electronic supplementary material

Below is the link to the electronic supplementary material.

Supplementary Material 1

Supplementary Material 2

Supplementary Material 3

Supplementary Material 4

## Data Availability

The datasets used and/or analyzed during the current study are available from the corresponding author upon reasonable request.
