# Supplementary material for: Development and validation of impact of early integration of palliative care and oncology(IEI PCO) questionnaire: a survey for medical oncologists and nurses
Source: BMC Palliat Care. 2024 Apr 26;23:109. doi: 10.1186/s12904-024-01435-1 (PMC11046835; doi:10.1186/s12904-024-01435-1)
Supplement: Supplementary file 1 — Supplementary Material 1 [file 12904_2024_1435_MOESM1_ESM.pdf]

Appendix (1) Criteria of Referral to Palliative Care V2

The criteria for patient referral to inpatient palliative-care consultation:(1-4)

1) Any Cancer Patient With Moderate To Severe Symptom regardless cancer stage.
2) Stage III Lung Or Pancreatic Cancer
3) Stage IV Cancer
4) Prior Hospitalization Within 30 Day (Excluding Routine Chemotherapy)
5) Hospitalization For Longer Than 7 Days;
6) Uncontrolled Symptoms, Including Pain, Nausea, Vomiting, Dyspnoea, Delirium, And Psychosocial Distress.
7) Decreased Eastern Cooperative Oncology Group (ECOG) performance status ≥3
8) The presence of one or more serious comorbid diseases that are also associated with poor prognosis.
9) The presence of complex care requirements (e.g. functional dependency; complex home support for antibiotics or feedings)
10) The presence of complex situation of relatives, e.g. small children involved, functional dependence and acute illness of primary care provider.

Identifying patients with unmet palliative-care needs who would benefit from an outpatient consultation. The criteria for referral to an outpatient consultation include: (5-7)

a) In the context of early integration; it is always advised to refer any cancer patient from day 1 diagnosis to palliative care OPD.
b) the presence of metastatic or locally advanced cancer;
c) Any Cancer Patient With Moderate To Severe Symptom regardless cancer stage.
d) Decreased Eastern Cooperative Oncology Group (ECOG) performance status.
e) The presence of one or more serious complication of advanced-stage cancer that is associated with a prognosis of less than 12 months.
f) The presence of one or more serious comorbid diseases that are also associated with poor prognosis.
g) The presence of complex situation of relatives, e.g. small children involved, functional dependence and acute illness of primary care provider.
h) Repeated ER visits

Generally palliative-care referral tailored to the local healthcare system and resource availability. (7)

References:

1) Weissman DE, Meier DE. Identifying patients in need of a palliative care assessment in the hospital setting: a consensus report from the Center to Advance Palliative Care. Journal of Palliative Medicine. 2011; 14:17–23. [PubMed: 21133809]
2) Center to Advance Palliative Care. Center to Advance Palliative Care; New York: 2015. https://www.capc.org/Last Last accessed: October 6, 2015
3) Adelson K, Paris J, Smith CB, Horton J, Morrison SR. Standardized criteria for required palliative care consultation on the solid tumor oncology service. American Society of Clinical Oncology Quality Care Symposium. 2013 Abstact 37 (J Clin Oncol, San Diego).
4) Gärtner J, Daun M, Wolf J, von Bergwelt-Baildon M, Hallek M. Early Palliative Care: Pro, but Please Be Precise! Oncol Res Treat. 2019;42(1-2):11-18. doi: 10.1159/000496184. Epub 2019 Jan 26. PMID: 30685764.
5) Glare P, et al. Study using the NCCN guidelines for palliative care to screen patients for palliative care needs and referral to palliative care specialists. J Natl Compr Canc Netw. 2013; 11:1087–96. [PubMed: 24029124]
6) Glare PA, Semple D, Stabler SM, Saltz LB. Palliative care in the outpatient oncology setting: evaluation of a practical set of referral criteria. J Oncol Pract. 2011; 7:366–70. [PubMed: 22379418]
7) Glare PA, Chow K. Validation of a Simple Screening Tool for Identifying Unmet Palliative Care Needs in Patients With Cancer. J Oncol Pract. 2015; 11:e81–86.
8) Hui D, Bruera E. Integrating palliative care into the trajectory of cancer care. Nat Rev Clin Oncol. 2016 Mar;13(3):159-71. doi: 10.1038/nrclinonc.2015.201. Epub 2015 Nov 24. PMID: 26598947; PMCID: PMC4772864.
