# Supplementary material for: Development and validation of impact of early integration of palliative care and oncology(IEI PCO) questionnaire: a survey for medical oncologists and nurses
Source: BMC Palliat Care. 2024 Apr 26;23:109. doi: 10.1186/s12904-024-01435-1 (PMC11046835; doi:10.1186/s12904-024-01435-1)
Supplement: Supplementary file 2 — Supplementary Material 2 [file 12904_2024_1435_MOESM2_ESM.pdf]

Appendix (2): Impact of Early Integration of Palliative Care and Oncology (IEI PCO survey)

Participant number:

Section I: Socio-demographic Data

1. Date of birth:……………………………
2. Sex: ………………………………
3. Qualifications:…………………
4. Occupation:………………………
5. Place of work (department/unit): ………………….
6. Working experience in oncology field (in years): ……………
7. How much of your practice involves the care of patients with advanced (incurable) cancer?
   a. None
   b. A small proportion
   c. A substantial proportion
   d. Most of my practice

Section II: Palliative Care Education

8. Have you ever had any training in PC during your oncology fellowship/ residency program?
   a) Yes
   b) No
9. If the answer to Q8 is yes, then how long was the training course?
   a) 1 week or less
   b) 1 month
   c) 6 weeks or more
10. What type of training/qualification completed in PC?
   a) Master
   b) Course
   c) Lectures
   d) Non
11. Was the training course relevant to your practice?
   a) Yes
   b) No
12. Do you agree that PC should be a part of postgraduate oncology training?
   a) Yes
   b) No

PC: Palliative Care

Section III: The Structure of clinical practice and the process of care

/6 =

1) Do you agree with the following statements about the structure of the process of care of PCO?

	Strongly disagree	Disagree	Don’t know	Agree	Strongly agree
a) All cancer centers must have PC services.					
b) Cancer patients should be seen by PMT even if they are on anti-tumor therapies.					
c) Integrating all units of oncology with PC services has great impact on overall patients' care and QoL.					
d) Process of PCO integration should take place in a structured way through departmental organizations, regular meeting and cases discussion					

e) Professional communication between oncology staff and PMT is essential for patient' care.					
f) Case discussion between PMT and oncologists increased oncologists' experience in holistic care.					

PC: Palliative Care, PMT: Palliative Medicine team, QoL: Quality of Life, PCO: Palliative Care and Oncology.

2) What are models for the delivery of simultaneous oncology and PC at your oncology center? **/8**
<u>(check all that apply)</u>

	Before integration	After integration
a) Inpatient PC consultation service		
b) Regular palliative care outpatient clinic		
c) On-demand joint oncology-palliative care outpatient-clinic		
d) Palliative care unit		
e) Weekends and holidays PMT inpatient ward round		
f) 24/7 phone calls for continuity of care		
g) Standalone PCC with 24h services		
h) Community-based palliative care or home health care		

PCC: Palliative Care Centre, PC: Palliative Care, PMT: Palliative Medicine team

3) **Regarding Inpatient Consultation Service**

I. Does your Centre have a dedicated PMT consultation service? 3. Yes 0. No

II. Is the patient seen in the same day upon consultation?

3. All of the time 2. Mostly
1. Rarely 0. No

/3

4) **Regarding Palliative Care Outpatient Clinic**

Is the patient seen in **same day in the OPD upon** demand of the oncologists?

3. All of the time 2. Mostly
2. Rarely 0. No

Regarding discharge planning and continuity of care **/4**

	Strongly disagree	Disagree	Don't know	Agree	Strongly agree
a) Adequate quantities of symptom control medications provided during discharge					
b) Follow-up plan provided during discharge					
c) After hours support provide					
d) Preferred place of care discussed and facilitated					

Section IV: Symptomatic Management /9

5) Do you agree with the following statements about the symptomatic management of cancer patients ***before*** and ***after*** PCO integration?

		Strongly disagree	Disagree	Don't know	Agree	Strongly agree
a) Physical, psychological, social and spiritual pain was properly managed	**Before**					
	**After**					
b) Dyspnea and other respiratory symptoms were easy to manage	**Before**					
	**After**					
c) Difficult cases of nausea and vomiting were well controlled	**Before**					
	**After**					
d) Constipation and other GIT symptoms were underestimated and under treated	**Before**					
	**After**					
e) Psychological issues (e.g. depression, insomnia and anxiety) were routinely assessed and properly managed	**Before**					
	**After**					
f) Delirium was easily identified and managed	**Before**					
	**After**					
g) Opioids initiation, titration, rotation and related side effects were properly managed	**Before**					
	**After**					
h) Symptoms were adequately controlled on discharge	**Before**					
	**After**					
i) Allowing for more effective delivery of oncological treatments through control of symptoms	**Before**					
	**After**					

GIT: gastrointestinal tract, PCO: Palliative Care and Oncology

Section V: Communication with Patients and Family /5

6) Do you agree with the following statements about communication with patients and family ***before*** and ***after*** PCO integration?

		Strongly disagree	Disagree	Don't know	Agree	Strongly agree
a) Repeated honest and accurate communication in a sensitive manner.	**Before**					
	**After**					
b) Goals of care were discussed.	**Before**					
	**After**					
c) Dealing more effectively with issues of ending active treatments.	**Before**					
	**After**					
d) Conflicts among patient, family and medical team were resolved	**Before**					
	**After**					
e) Higher patients' and families' acceptance of PC policy of transfer.	**Before**					
	**After**					

PCO: Palliative Care and Oncology

Section VI: End of Life Care

/7

7) Do you agree with the following statements about end-of-life care ***before*** and ***after*** the PCO?

		Strongly disagree	Disagree	Don't know	Agree	Strongly agree
a) End of life symptoms were effectively managed (e.g. delirium, pain, upper respiratory secretions)	**Before**					
	**After**					
b) Prognosis was communicated clearly to the family.	**Before**					
	**After**					
c) Compassionate communication was regularly delivered to patient, family and medical staff	**Before**					
	**After**					
d) Bereavement support was provided	**Before**					
	**After**					
e) Limitation of the role of life sustaining measures were discussed	**Before**					
	**After**					
f) Patient and family values, preferences and goals were discussed and incorporated into PC plan	**Before**					
	**After**					
g) Managing the place of death based on patient/family preference were discussed and declared (eg: ICU, home..)	**Before**					
	**After**					

PCO: Palliative Care and Oncology

Section VII: Work burden

/7

8) Do you agree with the following statements regarding work burden ***after*** PCO integration?

	Strongly disagree	Disagree	Don't know	Agree	Strongly agree
a) The length of oncologists' visits to patients during rounds is reduced					
b) Number of patients' calls are less					
c) Number of nurses' calls to the oncologists are less					
d) Number of patients' visits to causality are less					
e) Number of psychiatric and ICU consultations are less					
f) Duty hours became less stressful					
g) I became more confident in dealing with patients' symptoms					

PCO: Palliative Care and Oncology

Section VIII: Attitude /7

9) Do you agree with the following statements about the role of PC?

	Strongly disagree	Disagree	Don't know	Agree	Strongly agree
a) I likely to refer my patient to PMT when cancer is first diagnosed.					
b) I have an ethical obligation to provide EoL care to my patient with terminal cancer rather than PMT.					
c) I only refer my patient to PCC at the time of impending death					
d) Referring my patient to PMT makes me lose hope					
e) I believe the response of PMT to referrals is slow.					
f) I think the criteria of PC referral is so restrictive to meet my patient' needs.					
g) I believe there is a need to educate patients, caregivers and even healthcare providers about the potential benefits of PC					

PC: Palliative Care, PMT: Palliative Medicine team, EoL:End of Life, PCO: Palliative Care and Oncology, PCC: Palliative Care Center

Section IX: Satisfaction /7

11) To what extent are you satisfied with ….?

	Very dissatisfied	Dissatisfied	Neither satisfied nor dissatisfied	satisfied	Very satisfied
a) Availability of PC services					
b) Accessibility of PC services					
c) Acceptability of PC services					
d) Continuity of PC services					
e) Quality of PC services					
f) Cost impact of PC services					
g) The overall services provided by PMT					

12) Do you think that the PMT services ~~needs~~ need~~s~~ to be improved?

a) No

b) Yes (kindly indicate how……………………………………………………….........
……………………………………………………………………………………….....
....
....)

PC: Palliative Care, PMT: Palliative Medicine team
