# Supplementary material for: Development and validation of impact of early integration of palliative care and oncology(IEI PCO) questionnaire: a survey for medical oncologists and nurses
Source: BMC Palliat Care. 2024 Apr 26;23:109. doi: 10.1186/s12904-024-01435-1 (PMC11046835; doi:10.1186/s12904-024-01435-1)
Supplement: Supplementary file 3 — Supplementary Material 3 [file 12904_2024_1435_MOESM3_ESM.pdf]

Table (1S) Calculation of Item-Content Validity Items (I-CVI) for items of end-of-life care dimension during the first round of judgment

Items	Relevant (rating 3 or 4)	Not relevant (rating 1 or 2)	I-CVIs*	Interpretation
**1**	10	1	0.909091	Appropriate
**2**	6	5	0.545455	Not Appropriate
**3**	11	0	1	Appropriate
**4**	9	2	0.818182	Appropriate
**5**	8	3	0.727273	Need Revision
**6**	11	0	1	Appropriate
**7**	10	1	0.909091	Appropriate
**8**	9	2	0.818182	Appropriate
**9**	8	3	0.727273	Need Revision
**10**	7	4	0.636364	Not Appropriate

*Item-Content Validity Items, Number of experts=11, Interpretation of I-CVIs: If the I-CVI is higher than 79 percent, the item will be appropriate. If it is between 70 and 79 percent, it needs revision. If it is less than 70 percent, it is eliminated.

Table (2S) Calculating of Content Validity Ratio (CVR) for a sample of instrument items for items of symptomatic management dimension during the first round of judgment

Items	Ne*	CVR**	Interpretation
**1**	7	0.27272727	Eliminated
**2**	5	-0.09090909	Eliminated
**3**	10	0.81818182	Remained
**4**	9	0.63636364	Remained
**5**	6	0.09090909	Eliminated
**6**	8	0.45454545	Eliminated
**7**	4	-0.27272727	Eliminated
**8**	11	1	Remained
**9**	9	0.63636364	Remained
**10**	8	0.45454545	Eliminated

*** N_e :** Number of experts evaluated the item essential, **CVR**: content validity ratio
**CVR or Content Validity Ratio = (Ne-N/2)/(N/2) with 11 person at the expert panel (N=11), the items with the CVR bigger than 0.59 remained at the instrument and the rest eliminated.

Table (3S): Differences in symptom control, communication and end of life care between oncologists and nurses before and after palliative care and oncology (PCO) integration

		Doctors		Nurses		P Value	Total	
		Mean	SD	Mean	SD		Mean	SD
**Section IV: Symptom Control**	After	38.9231	4.55021	38.0382	4.38249	0.274	38.2412	4.42365
	Before	22.6667	6.74472	29.7786	8.22406	<0.001	28.1471	8.44100
	Diff	16.25641	7.54188	8.25954	9.11599	<0.001	10.09412	9.38540
	P value	<0.001		<0.001			<0.001	
**Section V: Communication skills**	After	21.6154	2.07257	21.4198	2.62811	0.629	21.4647	2.50714
	Before	15.0256	4.04245	17.7557	4.55410	<0.001	17.1294	4.57751
	Diff	6.58974	4.83800	3.66412	4.40386	<0.001	4.33529	4.65870
	P value	<0.001		<0.001			<0.001	
**Section VI: End of Life**	After	30.7436	3.53716	29.1450	2.64901	0.012	29.5118	2.94373
	Before	19.4103	5.06130	20.8855	5.42167	0.132	20.5471	5.36266
	Diff	11.33333	5.38191	8.25954	5.74535	0.003	8.96471	5.79522
	P value	<0.001		<0.001			<0.001	
