# Supplementary material for: Development and validation of impact of early integration of palliative care and oncology(IEI PCO) questionnaire: a survey for medical oncologists and nurses
Source: BMC Palliat Care. 2024 Apr 26;23:109. doi: 10.1186/s12904-024-01435-1 (PMC11046835; doi:10.1186/s12904-024-01435-1)
Supplement: Supplementary file 4 — Supplementary Material 4 [file 12904_2024_1435_MOESM4_ESM.pdf]

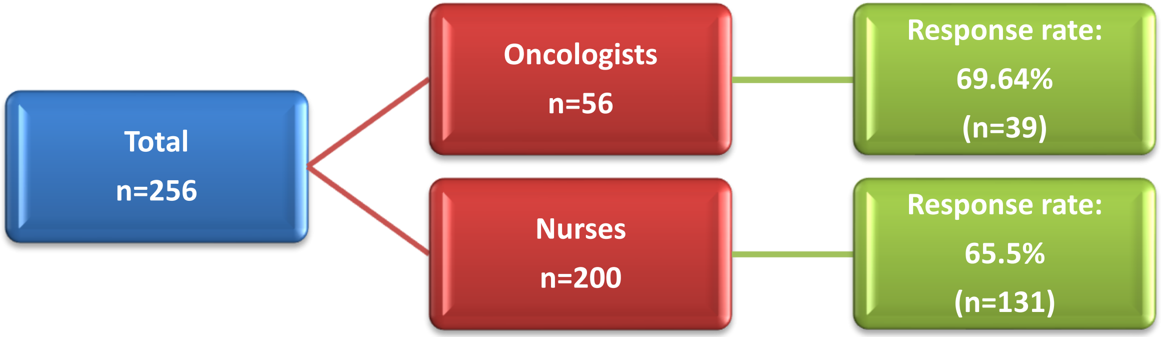

Figure (1S): Flowchart for distribution of Impact of Early Integration of Palliative Care and Oncology''(IEI PCO survey)

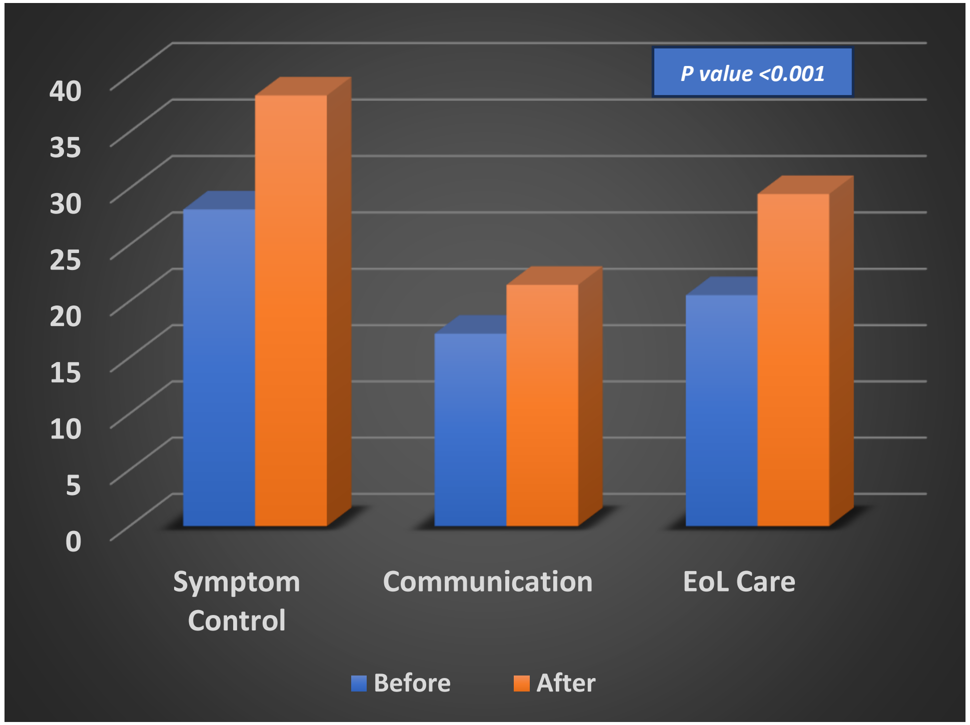

Figure (2S): Paired t-test Comparison between symptom control, communication and End of life care before and after PCO integration

EOL: End of life, PCO: Palliative Care Oncology
